# Effects of oxidative stress regulation in inflammation-associated gastric cancer progression treated using traditional Chinese medicines: A review

**DOI:** 10.1097/MD.0000000000036157

**Published:** 2023-11-17

**Authors:** Bo Chen, Xinqian Dong, Jinlong Zhang, Wei Wang, Yujiao Song, Xitong Sun, Kangning Zhao, Zhen Sun

**Affiliations:** a Shandong University of Traditional Chinese Medicine, Jinan, People’s Republic of China; b Affiliated Hospital of Shandong University of Traditional Chinese Medicine, Jinan, People’s Republic of China.

**Keywords:** gastric cancer, gastritis, natural prescriptions, NF-κB, Nrf2, oxidative stress

## Abstract

Gastric cancer (GC) is a global public health concern that poses a serious threat to human health owing to its high morbidity and mortality rates. Due to the lack of specificity of symptoms, patients with GC tend to be diagnosed at an advanced stage with poor prognosis. Therefore, the development of new treatment methods is particularly urgent. Chronic atrophic gastritis (CAG), a precancerous GC lesion, plays a key role in its occurrence and development. Oxidative stress has been identified as an important factor driving the development and progression of the pathological processes of CAG and GC. Therefore, regulating oxidative stress pathways can not only intervene in CAG development but also prevent the occurrence and metastasis of GC and improve the prognosis of GC patients. In this study, PubMed, CNKI, and Web of Science were used to search for a large number of relevant studies. The review results suggested that the active ingredients of traditional Chinese medicine (TCM) and TCM prescriptions could target and improve inflammation, pathological status, metastasis, and invasion of tumor cells, providing a potential new supplement for the treatment of CAG and GC.

## 1. Introduction

**Figure 1.**
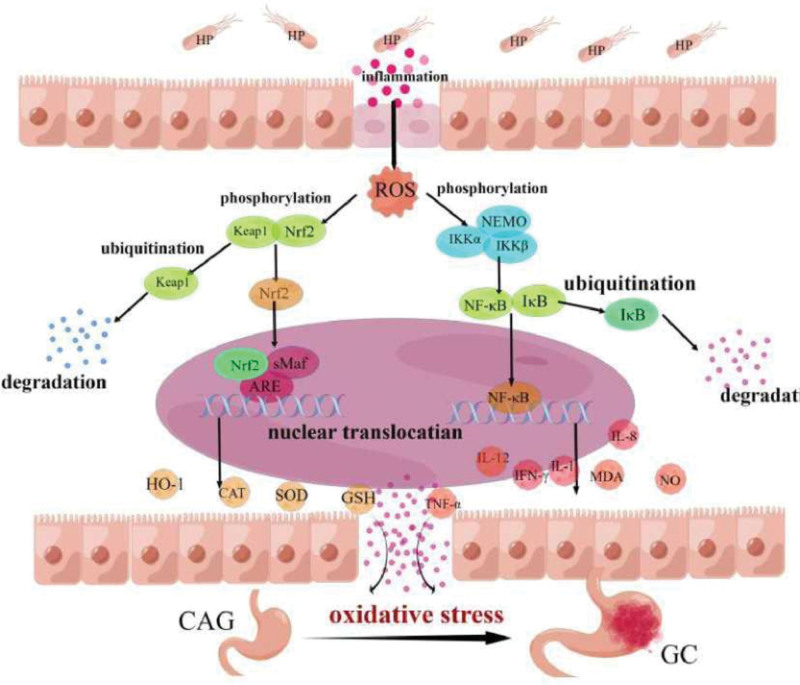
Biological processes of oxidative stress in gastric “inflammation-carcer”.

Gastric cancer (GC) is a common malignant disease of the digestive system that seriously threatens human health and poses an economic burden on families and society. Although the incidence of GC has significantly decreased, it remains the third most common cause of cancer-related deaths worldwide.^[1]^ With the rapid improvement in medical treatment, the treatment of GC is increasing; however, patient survival rates are still unsatisfactory, and the economic cost remains high. Therefore, reducing the incidence of GC is an issue that requires urgent resolution. Oxidative stress, as one of the important pathogeneses of chronic atrophic gastritis (CAG) and GC, promotes the development of the disease in many respects and actively participates in the inflammatory process, causing continuous damage to the gastric mucosa and ultimately leading to GC progression. Oxidative stress promotes CAG and GC progression through 2 processes: overexpression of oxidases, manifesting as an increase in reactive oxygen species (ROS), activation of the nuclear factor-kappa b (NF-κB) signaling pathway, accumulation of lipid peroxidation end products nitric oxide (NO) and malondialdehyde (MDA), and up-regulation of pro-inflammatory factors such as TNF-α, IL-I, and IL-8. These lead to aggravated gastric mucosal injury and increased pathological malignancy. Second, antioxidant enzyme deficiency meanss the physiological oxidative stress caused by oxidases cannot be counteracted, leading to inhibition of the nuclear factor erythroid 2-related factor 2 (Nrf2) signaling pathway, downregulation of superoxide dismutase (SOD), glutathione (GSH) and other antioxidant enzyme contents, and finally destruction of the cell DNA structure. Oxidative stress affects homeostasis of the endoplasmic reticulum, mitochondria, and other organelles, as well as normal cellular activity, induces carcinogenesis, and promotes tumor formation.^[2–4]^ An increasing number of studies have shown that oxidative stress can enhance the inflammatory expression of CAG, aggravate inflammatory infiltration of the gastric mucosa, and increase the risk of CAG carcinogenesis. In addition, the inhibition of oxidative stress damage to the gastric mucosa by reducing *Helicobacter pylori–*associated inflammation prevents subsequent GC development.^[5]^ Experiments have also verified that the upregulation of antioxidants exerts anticancer activity to prevent GC.^[6,7]^ The above studies showed that oxidative stress plays an important role in CAG and GC. Inhibition of oxidative stress can not only inhibit the occurrence of inflammation and hinder further carcinogenesis of CAG, but also intervene in GC by regulating this pathway (Fig. 1).

## 2. Observations

### 2.1. The role of oxidative stress in the course of gastric “inflammation carcinoma”

#### 2.1.1. MDA, SOD, and GSH as important biomarkers in gastric “inflammation carcinoma.”

MDA is a cytotoxic product formed by lipid peroxidation in the presence of oxygen free radicals which can react with proteins and nucleic acids, destroying cell membrane structure and function, changing membrane permeability, and participates in crosstalk with oxidative stress pathway target proteins to disrupt the redox balance and activate inflammatory factor and pro-oncogene expression, thus accelerating the progression of a variety of tumors (e.g., thyroid, lung, breast, and colon cancers).^[8–10]^ It has been reported that oxidative stress damage is more severe with higher MDA content.^[11]^ It has been found that *H pylori* (Hp) acts as a pro-inflammatory mediator, inducing DNA methylation, activating oxidative stress pathways, upregulating MDA content, and damaging the gastric mucosa leading to transformation of gastric “inflammation-carcinoma.”^[12]^ Notably, in patients with CAG and patients with intestinal metaplasia MDA content is significantly increased compared with that in healthy patients, and pathological changes are positively correlated with gastric mucosal injury.^[13,14]^ Liu et al found that oxidative stress could be significantly alleviated and gastric mucosal injury could be reduced by inhibiting MDA expression.^[15]^ It has been reported that there are higher MDA levels in GC cells and higher MDA levels diminishes the antioxidant capacity of the body. Normal gastric tissue cannot then resist the infiltration of cancer cells, resulting in extensive spread of cancer cells in the gastric wall and accelerated tumor progression.^[16]^ The above studies showed that an increase or decrease in MDA levels promotes or inhibits disease progression and that MDA plays an important role in measuring the level of oxidative stress. MDA levels can indirectly reflect the degree of gastric mucosal injury, be used as an evaluation index for disease development, and at the same time assess GC prognosis.

Glutathione (GSH) is a small peptide consisting of glutamate, cysteine, and glycine, which exists in both reduced (GSH) and oxidized (GSSG) forms.^[17,18]^ As an important antioxidant in cells, GSH mediates the regulation of target signaling molecule expression to maintain redox homeostasis and protect host cells from damage including damage caused by lipid peroxidation.^[19]^ GSH is oxidized to GSSG by glutathione peroxidase, while oxidative stress metabolites are reduced to nontoxic hydroxyl moieties, which in addition rapidly metabolize and decompose the resulting harmful substances and avoid the destruction of intact cell membrane structure and function by oxidative stress, and this effective defense is attributed to the actions of GSH via synthesis and redox pathways.^[20,21]^ Matsuoka detected gastric epithelial cells in patients with CAG and GC infected with Hp, and the results showed that GSH levels decreased to different extents in different patients, but decreased most significantly in patients with GC, indicating that GSH expression in the stomach was positively correlated with the degree of carcinogenesis of gastric epithelial pathology and that gastric mucosal injury was more severe in patients with GC.^[22]^ It has been reported that the virulence protein factor CagA secreted by Hp enables the nuclear translocation of transcriptional regulatory proteins to antagonize the regulation of GSH antioxidant enzymes.^[23]^ Velmurugan et al found that improving antioxidant capacity, such as that of GSH, inhibited oxidative stress damage to the gastric mucosa of atrophic gastritis model rats.^[24]^ Chen et al found that GSH can promote metabolic remodeling of cells to inhibit GC cell metastasis.^[25]^ The above studies showed that CAG and GC progression was related to the decrease of GSH antioxidant enzymes. Increasing GSH content could effectively inhibit the persistent damage caused by ROS and other oxides to the gastric mucosa, reduce the risk of carcinogenesis, and effectively inhibit GC metastasis and recurrence.

Superoxide dismutase is an antioxidant metalloprotease naturally found in organisms.^[26]^ It performs its important antioxidant role by effectively removing ROS from the body and protecting cells from ROS-induced oxidative damage.^[27]^ SOD is a key enzyme involved in free radical detoxification in the body. Under normal physiological conditions, ROS decompose to generate oxygen (O_2_) and hydrogen peroxide (H_2_O_2_). Meanwhile, H_2_O_2_ generates O_2_ and H_2_O through the enzymatic action of catalase (CAT).^[28]^ If the SOD content decreases or is insufficient, the scavenging ability of free radicals decreases, which can lead to the accumulation of a large number of peroxidation products leading to damage to normal mucosal tissues. Therefore, the upregulation of SOD levels can strengthen the ability of the body for defense and repair of free radical damage.^[29]^ It has been reported that SOD regulation is an important factor affecting tumor progression and survival in conditions including various gastrointestinal diseases.^[30]^ Xiao demonstrated that increasing SOD content could improve the protection and repair ability of the gastric mucosa, alleviate the level of oxidative stress, and reduce the risk of pathological intestinal metaplasia and malignant transformation of the gastric mucosa.^[31]^ Another study showed that SOD2 polymorphisms were associated with an increased GC risk.^[32]^ The results suggest that SOD has potential value in monitoring the degree of inflammatory injury to the gastric mucosa and gastric tumor formation.

In summary, MDA, GSH, and SOD have been used as biomarkers to evaluate oxidative stress in a large number of in vitro and in vivo experiments and can indirectly reflect the degree of gastric mucosal injury and GC progression and prognosis. Therefore, we can effectively inhibit oxidative stress-induced damage to the gastric mucosa by inhibiting the overexpression of MDA and ROS and increasing the SOD and GSH levels, thus further inhibiting the progression of gastric “inflammation-carcinoma.” This would improve the prognosis of patients with GC, reduce the resistance to chemotherapeutic drugs, improve the overall response rate of treatment, and improve patient survival rates.

#### 2.1.2. NO as an important inflammatory mediator in the course of gastric “inflammation cancer.”

NO is a free radical produced by nitric oxide synthase (NOS) via the oxidative pathway.^[33]^ It is an important regulatory substance involved biological processes such as apoptosis, immunity, and oxidative stress.^[34]^ Normally, NO levels maintain normal activity to protect the mucosal barrier from damage by toxic metabolites. Under pathological conditions, elevated NO levels destroys the main components of cells, inhibits protein synthesis, damages DNA molecules, destroys mucosal integrity, and ultimately promotes carcinogenesis.^[35]^ In addition, when stimulated by inflammatory molecules, inducible nitric oxide synthase (iNOS) produces high concentrations of NO which promotes tumor angiogenesis and abnormal cell proliferation and inhibits the physiological apoptosis of cells.^[36]^ Notably, NO inhibits leukocyte proliferation, leading to tumor growth and spread.^[37]^ Differences in NOS concentrations therefore have the ability to promote or inhibit disease. Studies have shown that NO plays an important role in maintaining the integrity of the gastric mucosa and microvascular barrier function. Normal levels of NO can regulate gastric mucosal blood flow, enhance the recovery and defence abilities of the mucosa, and can improve the ability of the gastric mucosa to clear toxic substances and resist oxygen free radicals. Excessive NO levels activate oxidative stress pathways, up-regulate inflammatory levels, accelerate DNA mutations, change the normal cell architecture and function, reduce the self-clearance ability of gastric mucosa, and increase the risk of carcinogenesis.^[38,39]^ Liao et al observed higher levels of iNOS and NO-induced damage in patients with GC. In addition, some pickled meats, fumigated products, ham, and other food products can increase endogenous NO production, cause lipid peroxidation, and promote GC formation.^[40,41]^ Studies also report that the downregulation of NO metabolite concentration in gastric juice could effectively reduce the degree of inflammatory infiltration and atrophy of the gastric mucosa, improve the histopathological status, and downregulate oxidative stress levels.^[42]^

#### 2.1.3. Nrf2 and NF-κB as important signaling pathways in the course of gastric “inflammation cancer.”

Nrf2 is a potent transcriptional activator that is commonly expressed in various organs and tissues and is involved in the upregulation of antioxidant proteins, improvement of antioxidant capacity, and exertion of biological effects against cytotoxicity and oxidative damage.^[43,44]^ Physiologically, Nrf2 and cytoskeleton-associated protein (Keap1) are present in the cytoplasm as inactive dimers through the binding of the Neh2 region at the N-terminus, thus maintaining enzymes and antioxidants that protect and stabilize cells at basal expression levels.^[45]^ In pathological conditions, Nrf2 dissociates from Keap1 and translocates into the nucleus, where the Neh1 region forms a heterodimeric complex with proteins such as Maf which then binds to antioxidant response element promoter sites to initiate the expression of Nrf2 downstream target genes to resist deleterious stimuli.^[46,47]^ Numerous studies have shown that Nrf2 plays an important role in protecting the gastrointestinal tract against oxidative stress and chemical damage to the gastrointestinal mucosa.^[48]^ Yanaka demonstrated that activating Nrf2 expression could upregulate antioxidant enzyme activity, improve the antioxidant capacity of the body, and protect and repair the gastric mucosa from oxidative stress damage during Hp infection.^[49]^ Farkhondeh et al showed that Nrf2 reduces GC recurrence rates and drug resistance through the antioxidant pathway, which has significant implications for GC treatment.^[50]^ In addition, Zheng et al confirmed that Nrf2 is associated with GC infiltration and can be used as an indicator of prognosis.^[51]^ The above studies suggest that intervening in the expression of the key regulator Nrf2 can effectively inhibit the expression of upstream and downstream target molecules of the oxidative stress pathway and reduce the inflammatory response. Nrf2 expression may also be used to monitor the malignant transformation of disease, which has significant implications for the development of drugs using the Nrf2 antioxidant pathway to treat various malignant diseases.

Nuclear factor-κB (NF-κB) is another important transcriptional regulator whose family consists of 5 transcriptional regulators, each of which has an N-terminal Rel homology domain responsible for its binding to DNA as well as dimerization, which differentially impacts cellular activity.^[52,53]^ This signaling pathway can bidirectionally regulate the biological processes of cells, meaning it can both regulate the oxidative stress pathway to remove abnormal cells and inhibit neoplastic disease progression, but also promote abnormal proliferation and differentiation of cells, leading to malignant transformation of neoplastic diseases.^[54]^The NF-κB signaling pathway acts on cytokines in both classical and non-classical pathways to maintain orderly cell signaling. Inactive NF-κB is present in the cytoplasm in a complex that binds to the inhibitory protein IκB into a trimer (p50-p65-IκB), which binds to NF-κB dimers mainly in the cytoplasm and plays an important role in signaling responses.^[55,56]^ Classical signaling pathways are activated by IKK complexes (IKKβ, IKKα, and NEMO) that phosphorylate IκB when stimulated by cytokines including inflammatory factors (e.g., IL-Iβ) or tumor necrosis factor (e.g., TNF-α). Phosphorylated IκB dissociates from the trimeric complex, is degraded by the proteasome after ubiquitination modification, and releases the NF-κB/Rel complex. Activated NF-κB/Rel complexes translocate into the nucleus where they interact with other transcription factors to induce target gene expression.^[57,58]^ In the non-canonical signaling pathway, the NF-κB2p100/Rel B complex stays in the cytoplasm in an inactive state and activates the kinase NIK through receptors (e.g., LTβR). Activated NIK in turn activates the IKKα complex, which phosphorylates the carboxy-terminal amino acids of p100. Phosphorylated p100 is degraded to P52 after ubiquitination, eventually forming an active p52/Rel heterodimer complex that promotes the conduction of cell signals after transferring the nucleus.^[59,60]^ It has been shown that the activation of NF-κB pathway can promote the occurrence and development of CAG and GC.^[61]^ In addition, ROS induces NF-κB to promote IL-8 transcription in gastric epithelial cells, promoting the inflammatory response of the gastric mucosa, up-regulating oxidative stress and change the pathology of the gastric mucosa. Inhibition of Hp-induced NF-κB activation reduces inflammatory factor expression to counteract the effects of oxidative stress in the gastric mucosa and further inhibit gastric “inflammation-carcinoma” progression.^[23,62–64]^

The above studies have shown that oxidative stress plays a dual role in the course of gastric “inflammation-carcinoma.” Activation of oxidative stress pathways promotes the progression of inflammation associated gastric carcinoma, where inhibition of key oxidative stress pathways effectively halts this progression by ameliorating gastric mucosal injury. Currently, the therapeutic effect of Western medicine alone on CAG and GC is unsatisfactory. Recently, a large number of in vivo and in vitro experiments have demonstrated the therapeutic effects of traditional Chinese medicines (TCM), which often have multiple active components and drug targets and few adverse effects, which is advantageous in the treatment of diseases.

### 2.2. Active ingredients of Chinese herbs regulate oxidative stress and gastric “inflammation-cancer”

**Figure 2.**
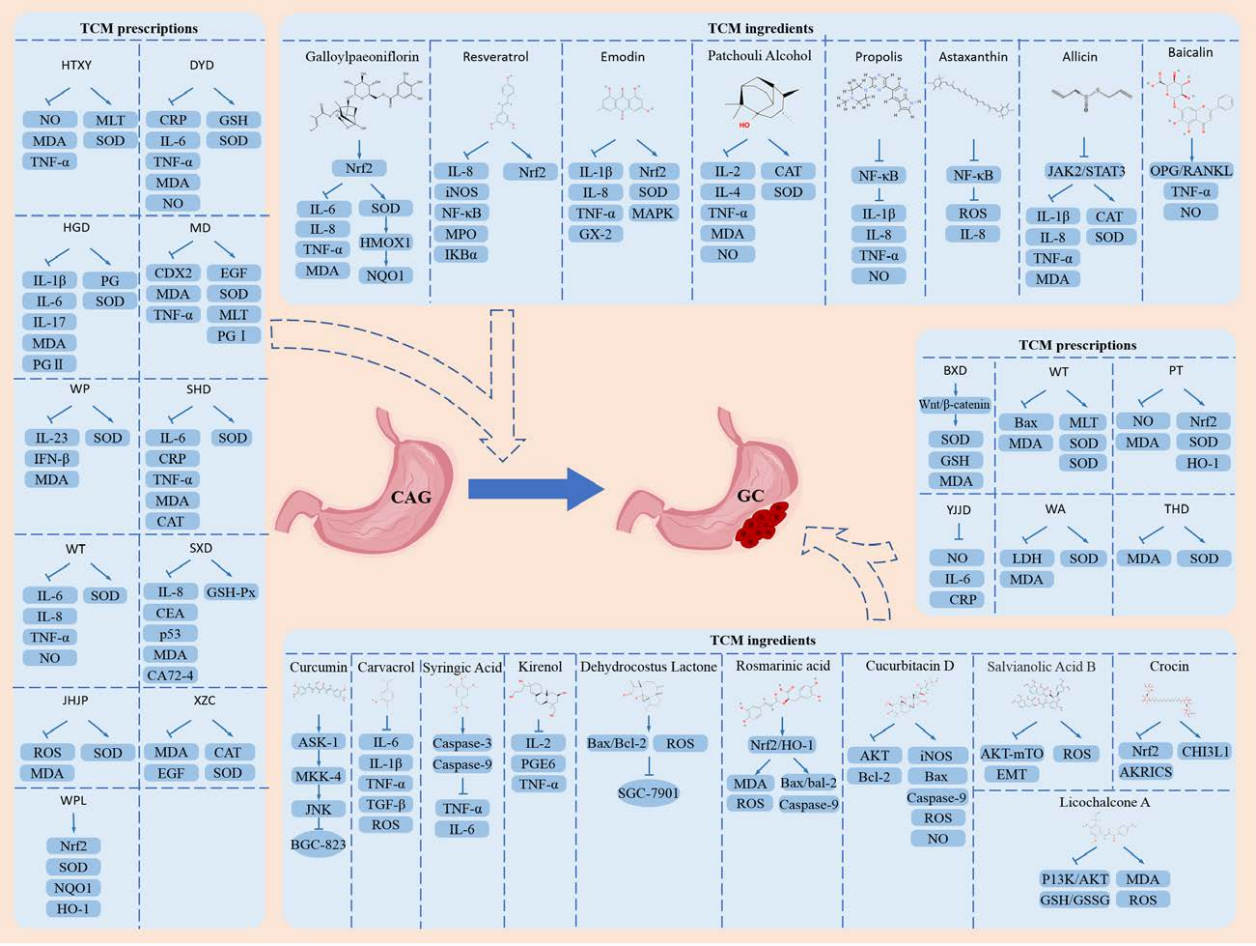
Mechanism diagram of active ingredients of traditional Chinese medicine and traditional Chinese medicine compound regulating oxidative stress to intervene gastric “inflammation-cancer”.

As mentioned earlier, NF-κB and Nrf2 signaling pathways are important transcriptional regulators and play a key role in the course of CAG. In addition, key marker proteins of the oxidative stress signaling pathway, such as MDA, SOD, and GSH, can also reflect the degree of gastric mucosal damage. With the development of molecular biology, network pharmacology, and other technologies, it is possible to reveal the pharmacological mechanism of a drug in the treatment of a disease at the molecular level. This provides a theoretical basis for the qualification and quantification of the effects of TCM in the treatment of diseases. An increasing number of active ingredients have been identified in TCM preparations, shedding light on new approaches for treating various cancers. They have prominent advantages such as a single composition and a clear mechanism of action, and play a significant role in the treatment of diseases. The following compounds are of positive significance in intervening in gastric “inflammation-carcinoma” progression by targeting and regulating NF-κB, Nrf2, and other signaling pathways and key proteins to inhibit oxidative stress. The data in this study were obtained from published articles; therefore, no ethical issues related to patients were involved. Therefore, approval from an ethics committee was not required (Fig. 2 and Table 1).

**Table 1:** Active ingredients in TCM in regulating oxidative stress in gastric “inflammation-carcer.”

Name	Models	Biological effects	Results	Pathway	References
Extract of Ginkgo Biloba	MNNG model rats	SOD↑	Inhibits oxidative stress and Inhibits oxidative stress and reduces the risk of gastric carcinogenesis	------	^[65]^
		MDA↓ Bcl-2↓ FasL↓			
Patchouli Alcohol	CEG-1 cells	SOD↑ CAT↑	Inhibits oxidative stress and reduces intracellular ROS	------	^[66]^
		NO↓ ROS↓ MDA↓ IL-4↓ TNF-α↓ IL-2↓			
Allicin	CAG model rats	SOD↑ CAT↑	Reducing gastric mucosal lesions, inhibiting oxidative stress and reversing intestinal chemotaxis in CAG rats	JAK2/STAT3 pathway	^[67]^
		MDA↓ IL-6↓ TNF-α↓ IL-1β↓			
Baicalin	CAG model rats	SOD↑ NO↑	Reduce the damage of gastric mucosa and increase the body weight of rats	OPG/RANKL pathway	^[68]^
Curcumin	BGC-823 cells	ROS↑	Promote apoptosis and inhibit the progression of gastric cancer	ASK1-MKK4-JNK pathway	^[69]^
Carvacrol	GC model rats	ROS↓IL-6↓ IL-1β↓ TNF-α↓ TGF-β↓	Mitigating oxidative stress and inflammatory infiltration to inhibit gastric cancer	-----	^[70]^
Galloylpaeoniforin	CEG-1 cells	SOD↑ Nrf2↑	inhibit oxidative stress and inflammatory factor production, reduce gastric mucosal damage	Nrf2 pathway	^[71]^
		MDA↓ IL-1β↓ ROS↓ TNF-α↓ IL-8↓			
Resveratrol	CAG model mouse	Nrf2↑ HO-1↑	Inhibition of oxidative stress and inflammation and improvement of gastric mucosal histopathological scores	NrF2/HO-1 pathway	^[72]^
		IL-8↓ iNOS↓ NF-κB↓ IκBα↓			
Red Ginseng Extract	CEG-1 cells	iNOS↓ ROS↓ NADPH↓ NF-κB↓ Jak1/ Stat2↓	Inhibition of oxidative stress	NF-κB/Jak1/ Stat2 pathway	^[73]^
Emodin	CAG model mouse	SOD↑	Significant improvement of gastric mucosal histopathology, inhibition of inflammation and oxidative stress	Nrf2/MAPK pathway	^[74]^
		IL-6↓ IL-1β↓ MDA↓ TNF-α↓			
Astaxanthin	AGS cells	ROS↓ IL-9662↓ IL-8↓ PPAR-γ↓	Effectively alleviating inflammatory infiltration	NF-κB pathway	^[75]^
Kirenol	MNG rats	mRNA↓ TNF-α↓ IL-2↓ PGE6↓	Suppresses inflammatory expression and reduces tumor incidence	NF-κB pathway	^[76]^
Crocin	AGS cells SGC-7901 cells	AKR1Cs↑	Enhances antioxidant capacity and exerts anti-cancer effects	Nrf2 pathway	^[77]^
		CHI3L1↓			
Aqueous Ginger Extract	MNU rats	SOD↑ GPx↑ GR↑	Inhibiting the progression of gastric cancer	NF-κB pathway	^[78]^
		TNF-α↓ IL-6↓			
Rosmarinic acid	SGC-7901 cells	ROS↑ MDA↑ Bax/Bcl-2↑	It promotes apoptosis of gastric cancer cells and exerts anticancer effects.	Nrf2/HO-1 pathway	^[79]^
Dehydrocostus Lactone	SGC-7901 cells	ROS↑ Caspase-3↑	Induction of apoptosis in gastric cancer cells to intervene in gastric cancer progression	Bax/Bcl-2 pathway	^[80]^
		Beclin1↓ LC3 I/LC3 II↑			
Cucurbitacin D	AGS cells SNU1 cells Hs2T cells	ROS↑ NO ↑ Bax↑ Caspase-9↑	Accelerating gastric cancer cell apoptosis and intervening in gastric cancer progression	AKT pathway	^[81]^
		Bcl-2↓			
Syringic Acid	AGS cells	Caspase-3↑ Caspase-9↑	Enhances antioxidant capacity, down-regulates inflammation levels and inhibits gastric cancer progression	mTOR/AKT pathway	^[82]^
		IL-6↓ TNF-α↓			
Licochalcone A	BGC cells	MDA↑ ROS↑	Inhibits the activity of gastric cancer cells	PI3K/AKT pathway	^[83]^
		GSH/GSSG↓			
Salvianolic acid B	AGS cells AGS/DDP cells	ROS↑	Reducing drug resistance rate and inhibiting gastric cancer progression	AKT/mTOR pathway	^[84]^
		EMT↓ NF-kB↓ TNF-a↓ IL-6↓ PGE↓			

CAG = chronic atrophic gastritis, CAT = catalase enzyme, GPX = glutathione peroxidase, GSH = glutathione, MDA = malondialdehyde, NF-κB = nuclear factor-Κb, iNOS = inducible nitric oxide synthase, Nrf2 = nuclear factor erythroid 2-related factor 2, ROS = reactive oxygen species, SOD = superoxide dismutase, TCM = traditional Chinese medicine.

#### 2.2.1. Pathological damage to gastric mucosa decreased through regulation of ROS, MDA, SOD, and GSH.

The extract of *Ginkgo biloba* (EGb) is a natural plant component and nutraceutical derived from the leaves of *Ginkgo biloba* with antioxidant and blood circulation-boosting effects.^[85]^ Previous studies have shown that EGb regulates neurotransmitters, delays neuronal degeneration, improves vascular microcirculation and body tolerance.^[86,87]^ Jiang et al treated N-methyl-N’-nitro-N-nitrosoguanidine (MNNG)-treated rats with EGb by gavage to observe EGb effects in an animal model of gastric cancer.^[65]^ The results showed that the extract enhanced SOD activity, inhibited MDA expression in a dose-dependent manner, and downregulated the expression of *c-myc, bcl-2*, and *fasL* gastric mucosal oncogenes. EGb may exert its actions via a reduction in the degree mucosal inflammation, intestinal metaplasia, and gastric mucosal dysplasia by regulating cell proliferation and apoptosis and upregulating antioxidant activity, which inhibits the progression of precancerous gastric lesions. EGb is therefore a potential therapeutic agent for CAG treatment. Patchouli is the dried aerial part of *Pogostemon cablin* (Blanco) Benth. Owing to its unique aroma and ability to treat various diseases, it is widely used in cosmetics and medicine.^[88]^ Patchouli alcohol (PA), a sesquiterpenoid, is an important component of the essential oils extracted from patchouli plants.^[89]^ Modern pharmacological studies have shown that PA has good antioxidant, analgesic, anti-inflammatory, and antidepressant effects, as well as multiple biological activities.^[90]^ Previous studies have shown that PA can inhibit Hp activity and exert antioxidant effects during CAG treatment.^[91]^ Xie et al found that PA inhibited ROS production, decreased the expression levels of inflammatory factors such as TNF-α, IL-2, and IL-4, up-regulated CAT and SOD expression level, and down-regulated the MDA and NO contents in gastric epithelial cells (GES-1) cultured with *Helicobacter pylori* urease.^[66]^ This suggests that PA protects against *H pylori* urease-induced GES-1 cell injury. Its protective mechanism may be related to the attenuation of apoptosis, reduction of intracellular ROS, and improvement of antioxidant and anti-inflammatory capacities, which have potential implications for PA-mediated oxidative stress reduction for the treatment of CAG. Allicin, an active compound derived from garlic, has anti-inflammatory, antioxidant, and anticancer effects.^[92]^ Chen et al found that allicin improved the pathological state of intestinal metaplasia and restored blood flow in rat gastric mucosa. In addition, it also downregulated the expression of TNF-α, IL-6, IL-1β, and MDA and increased SOD and CAT activities. This may be related to the inhibition of the JAK2/STAT3 signaling pathway activation by allicin.^[67]^ Baicalin (BI) is an active flavonoid isolated from TCM *Scutellarin baicalinase*, which has outstanding anti-inflammatory, antioxidant, and immunomodulatory effects.^[93]^ Niu demonstrated that BI had a protective effects on the gastric mucosa of CAG model rats and could promote the serum expression of NO and SOD, promote CAG cell apoptosis, and relieve oxidative stress. This mechanism was related to the activation of the OPG/RANKL axis.^[68]^ Curcumin is an active substance extracted from the rhizome of *Curcuma longa* and has demonstrated antitumor, antioxidant, and antiproliferative effects.^[94]^ Liang et al demonstrated the effects of curcumin on BGC-823 human gastric cancer cells, showing that curcumin induced the ROS-mediated ASK1-MKK4-JNK cascade and led to apoptosis in BGC-823 cells, which is of potential significance in the treatment of GC.^[69]^ Carvacrol is a monoterpene phenol produced by abundant aromatic plants and is used as a food and cosmetic additive. Modern pharmacological studies have shown that carvacrol exerts anti-inflammatory, antioxidant, and anticancer effects.^[95]^ Gunes et al investigated the gastric mucosal protective effect of carvacrol in an MNNG-induced GC rat model, and found that carvacrol effectively reduced the levels of pro-inflammatory factors such as IL-6, IL-1β, TNF-α, and TGF-β, reduced ROS activity, downregulated oxidative stress, and reduced the extent of inflammatory infiltration in the gastric mucosa of model rats, thus inhibiting GC progression.^[70]^

#### 2.2.2. Regulation of NF-κB, Nrf2 signaling pathways prevents gastric “inflammation-carcinoma” transformation.

Galloyl paeoniflorin (GPF) is an important chemical component isolated from part of the peony root. Modern pharmacological studies have confirmed that GPF has medicinal value due to its anti-inflammatory, antioxidant, and antitumor effects. It is widely used in the treatment of various diseases such as osteoporosis, cerebral ischemia, and allergic asthma.^[96–100]^ A recent study found that GPF could treat CAG and reduce Hp–induced gastric mucosal injury and pathological scores.^[71]^ The mechanism is related to activating the expression of Nrf2 and its downstream target genes to counteract oxidative stress and reduce the expression of pro-inflammatory factors such as TNF-α, IL6, and IL8, while upregulating SOD, HMOX1, and NQO1 levels and reducing MDA levels. Resveratrol is one of the most intensively studied and widely used polyphenols, and is present in a variety of plants.^[101]^ Evidence suggests that resveratrol has pharmacological effects including anti-inflammatory, anticancer, antioxidant, antiaging, and cardioprotective properties.^[102]^ Among these numerous biological effects, its antioxidant properties are the most prominent. It exerts these effects by scavenging or inhibiting the synthesis of oxygen free radicals, inhibiting lipid peroxidation, regulating the activity of related antioxidant enzymes, and stabilizing the oxidation-antioxidant balance, leading to beneficial effects in CAG.^[103,104]^ Resveratrol relieves gastric mucosal injury by down-regulating IL-8, iNOS, and MPO levels, thus inhibiting IκBα phosphorylation, blocking NF-κB activation, increasing the expression levels of Nrf2 and HO-1, and reducing the degree of gastric mucosal injury in Hp-induced gastritis mice. This suggests that the antioxidant mechanism of resveratrol may be related to the Nrf2 and NF-κB pathways.^[72]^ Red ginseng is widely consumed as a traditional health-promoting food, while also playing an important role in the pharmaceutical field.^[105]^ Ginsenosides are the active components in red ginseng and have numerous biological effects, such as improving microcirculation and immunity and possess anti-inflammatory, anti-viral, and anti-oxidant properties.^[106]^ Cho et al investigated the effect of red ginseng extract (RGE) on Hp-induced human gastric epithelial cells (AGS) and showed that RGE inhibited oxidative stress in Hp-infected AGS cells and reduced gastric mucosal injury. Its mechanism of action may be that RGE downregulates NADPH oxidase, ROS production, and IL-8 expression by inhibiting the NF-κB pathway and blocking JAK 1/STAT 2 activation. RGE significantly improved the pathological state of AGS cells by regulating the expression of related cytokines, thereby preventing gastric epithelial cell carcinogenesis in a time-and dose-dependent manner.^[73]^ Rhubarb is recorded in Shennong Ben Cao Jing and is the dried root and rhizome of *Rheum officinale, R. palmatu*, and *R. tanguticum*, medicinal plants of the Liao family.^[107]^ Because it has purging effects such as, clearing heat, purging fire, cooling and detoxifying blood, reducing blood stasis, unblocking meridians, and removing dampness and yellowing, it has a therapeutic effect in digestive, endocrine, cardiovascular, and malignant diseases.^[108]^ Studies have shown that rhubarb extracts and monomeric prescriptions have excellent anti-inflammatory activities, mainly through the regulation of biological processes such as oxidative stress, apoptosis, and immune responses.^[109,110]^ Liu et al found that in CAG model mice, the levels of pro-inflammatory factors TNF-α, COX-2, IL-6, and IL-1β, MDA content was increased, and SOD expression was downregulated, indicating that the decrease in antioxidant enzymes in the gastric mucosa. This may have been caused by the inhibition of the Nrf2 and MAPK signaling pathways.^[74]^ After intervention with emodin, the Nrf2 and MAPK signaling pathways were activated, the expression of inflammatory protein factors was inhibited, and antioxidant enzyme activity was increased to protect the structural integrity of the gastric mucosa. This revealed the molecular mechanism by which emodin prevents oxidative stress-induced damage to the gastric mucosa, providing a further evidence for the clinical application of this drug in diseases affecting the gastric mucosa. Propolis is a natural resin extracted from various plants with anti-inflammatory, antioxidant, and anticancer activities. Song et al found that gastric mucosal injury was significantly improved in CAG model mice treated with propolis, accompanied by downregulated inflammatory factors (IL-8, TNF-α, and IL-1β) and decreased NO expression. Western blotting analysis results showed that propolis also significantly inhibited IκBα and p65 phosphorylation, thereby inhibiting p65 translocation from the cytoplasm to the nucleus to reduce expression of pro-inflammatory target genes in model mice. Its therapeutic mechanism is related to inhibition of NF-κB signaling pathway and alleviation of oxidative stress.^[111]^ Astaxanthin, a naturally fat-soluble carotenoid, can be extracted from various seafoods and is used in the treatment of various diseases owing to its antioxidant, anticancer, DNA repair, and anti-apoptotic properties.^[112]^ Kim et al found in AGS cells infected with Hp that treatment with astaxanthin significantly inhibited ROS activity, decreased IL-8 expression, and blocked NF-κB pathway activation.^[75]^ This suggests that astaxanthin can alleviate oxidative stress and inflammatory damage to the gastric mucosa and effectively alleviate inflammatory infiltration. Kirenol has been found to be a natural diterpenoid with anti-inflammatory and anticancer properties.^[113]^ Kirenol downregulated lipid peroxidation, increased enzymatic and nonenzymatic antioxidants, suppressed TNF-α, IL-2, and PGE6 expression, and reduced tumor incidence in MMNG rats via an action mechanism related to kirenol inhibition the of NF-κB signaling pathway.^[76]^ Crocin is a carotenoid extracted from the medicinal plant, saffron. Previous studies have shown that crocin can be used as a protective agent against gastric mucosal injury. In addition, crocin has lower toxicity and a better safety profile than the anticancer agent 5-fluorouracil (5-FU).^[114]^ Luo et al treated GC cell lines AGS and SGC-7901 using crocin. The results showed that crocin exerted anticancer effects by activating the classical Nrf2 signaling pathway and improving antioxidant capacity. Ginger is a common TCM that is widely used because of its anti-inflammatory, anticancer, and antioxidant effects and pharmacological activity in immune disorders.^[77,115]^ Mansingh et al found that aqueous ginger extract (AGE) was able to upregulate SOD, glutathione peroxidase and GR contents, decrease TNF-α and IL-6 expression, and alleviate oxidative stress and inflammatory factor expression associated with GC in N-methyl-N-nitrosourea (MNU)-treated rats, and its mechanism of action was associated with NF-κB.^[78]^ Rosmarinic acid (RA) is a phenolic compound extracted from plants such as rosemary and mint. It has anti-inflammatory, anti-cancer, antioxidant, and other pharmacological properties.^[116]^ Yang et al used gastric cancer cells (SGC-7901) to demonstrate that rosmarinic acid exerts anticancer activity by regulating the Nrf2/HO-1 related signaling pathways, increasing ROS and MDA activities, and activating oxidative stress to promote gastric cancer cell apoptosis.^[79]^

#### 2.2.3. Regulating other signaling pathways to intervene in GC progression.

Dehydrocostus lactone (DHCL) is derived from the dried roots of *Aucklandia lappa* Decne. It is a member of the family Asteraceae and has anti-inflammatory and antitumor effects.^[117]^ Shao et al showed that DHCL inhibits SGC-7901 proliferation, promotes intracellular ROS production, activates the Bax/Bcl-2 mediated mitochondrial apoptosis pathway, and induces GC cell apoptosis in a dose-dependent manner to inhibit GC progression.^[80]^ Cucurbitacin D (CuD) is a tetracyclic triterpenoid extracted from members of the family Cucurbitaceae, and various studies have shown that it has anti-inflammatory, hepatoprotective, and antitumor activities.^[118]^ Zhang et al showed that CuD could upregulate ROS expression and increase NO content in human gastric cancer cell lines (AGS, SNU1, and Hs2T), while also triggering apoptosis pathways, leading to higher ROS and NO production, thereby accelerating gastric cancer cell apoptosis.^[81]^ This inhibitory effect of CuD on GC progression was achieved by inducing iNOS/NO signaling and inhibiting the Akt pathway. Syringic acid (SA) is a natural phenolic compound present in herbs and edible plants and has anti-inflammatory, anti-oxidative, and anti-angiogenic effects.^[119]^ Pei et al found that SA could effectively inhibit GC progression through targeted inhibition of mTOR/AKT signaling pathway, significantly reduce p53 and Bcl -2 expression, enhance antioxidant capacity through mitochondrial membrane potential, up-regulate caspase-3 and caspase-9 activities, and down-regulate IL-6 and TNF-α levels.^[82]^ Licochalcone A (LA), a flavonoid derived from the root of *Glycyrrhiza uralensis*, has anti-inflammatory, anticancer, and lipid- and blood glucose-regulatory effects.^[120]^ Hao et al found that LA upregulates MDA expression, decreases the GSH/GSSG ratio, increases ROS activity, and inhibits GC cell proliferation.^[83]^ Furthermore, LA induces ROS-mediated MAPK activation, inhibits the PI3K/AKT signaling pathway, and prevents GC progression. Salvianolic acid B (Sal-B) is the active substance of the medicinal plant *Salvia miltiorrhiza* Bunge. Related studies have shown that Sal-B has anti-inflammatory, anticancer, and anti-fibrotic effects and promotes stem cell proliferation and differentiation.^[121]^ In addition, Wang et al treated AGS and AGS/DDP cells with Sal-B and found that Sal-B not only initiated ROS activity and promoted apoptosis but also reduced migration, invasion, and epithelial-mesenchymal transition (EMT) in AGS and AGS/DDP cells and reduced DDP resistance in GC cells, which was achieved via regulation of the AKT/mTOR pathway.^[84]^

The above studies have shown that various active ingredients can not only inhibit the inflammatory response by regulating the Nrf2, NF-κB, JAK1/STAT2 and MAPK signaling pathways, effectively reduce the degree of pathological damage caused by oxidative stress, but also protect the integrity of gastric mucosal structure, promote gastric mucosal self-repair, attenuate the progression of CAG, and finally reduce the rate of gastric mucosal carcinogenesis. These results have significance for guiding the study of active ingredients for targeted and precise treatment of CAG. Therefore, they are potential drugs for patients with gastric mucosal injuries. In addition, we found that oxidative stress is inextricably related to the progression, prognosis, and drug resistance of GC and inhibition or promotion of this process can have a clinical on GC development and progression. Therefore, in-depth studies should be conducted to increase the selectivity of natural prescriptions for GC treatment.

### 2.3. Chinese herbal prescription regulates oxidative stress and gastric “inflammation-cancer” transformation

#### 2.3.1. Increasing antioxidant factor expression and hindering GC development.

Compared with conventional drug active ingredients, Chinese herbal preparations have the overall characteristics and unique advantages of multiple active components and multiple targets with actions that may be exerted via multiple pathways. Many studies have reported that Chinese herbal preparations are effective for the CAG treatment. Liu et al treated CAG patients using Huatan Xiaoyu Fang (tangerine peel, *Pinellia ternata*, Ji Nei Jin, Danshen, Coix seed, Cat claw grass, Puhuang, *Scutellaria barbata*, and *Agrimonia pilosa*) and found that this formula could resist oxidative stress damage and improve pathological tissue lesions by downregulating MDA and TNF-α and increasing SOD and serum epidermal growth factor levels in the body.^[122]^ Dendrobium Yangwei Decoction (fried malt, fried licorice, Zhuru, fried Radix Paeoniae Alba, Beisha, *Trichosanthes kirilowii, Ophiopogon japonicus*, and *Dendrobium officinale*) activates blood circulation and relieves pain, nourishes yin, a nourishes the stomach meaning it is widely used in the treatment of CAG.^[123]^ Wu et al found in an in-depth study that in the treatment of patients with CAG this preparation can reduce the levels of C-reaction protein, IL-6, TNF-α, NO, and MDA, up-regulate the contents of GSH and SOD in the gastric mucosa, significantly improve inflammatory injury, effectively relieve oxidative stress, and inhibit the progression of the course of gastric “inflammation-carcinoma.”^[124]^ Zhu found that Huagan decoction (Radix Bupleuri, Pericarpium Citri Reticulatae, Cortex Moutan, Fructus Gardeniae, Fritillaria thunbergii, Radix Paeoniae Alba, Rhizoma Coptidis, Fructus Evodiae, Rhizoma Alismatis, and Radix Glycyrrhizae) combined with folic acid improved serum PG I and SOD levels and the function and antioxidant capacity of gastric mucosal glandular cells.^[125]^ Previous studies have confirmed that weipiling (Radix Pseudostellariae, Rhizoma Atractylodis Macrocephalae, Poria cocos, Radix Notoginseng, Rhizoma Curcumae, Herba Hedyotis diffusa, Rhizoma Monkey Mushroom, Shougong) can reverse precancerous lesions, such as CAG, and intestinal metaplasia and dysplasia.^[126]^ Pan et al used intragastric administration of Weipiling 3.75 g/kg and 15 g/kg to gastric precancerous lesion (GPL) model mice for 8 weeks and found that this decoction could increase the protein expression of SOD, up-regulate Nrf2, NQO1, and HO-1 expression, exert anti-oxidative stress injury in a dose-dependent manner, improve gastric mucosal lesions, reduce histological scores, and increase mouse body weight.^[127]^ These results suggest that this decoction exerts effects through the Nrf2/NQO1/HO-1 signaling pathway to control or even reverse gastric “inflammation-carcinoma” transformation and restore the damaged gastric mucosa. Zeng et al used Jianpi Huayu Jiedu decoction (which includes *Hericium erinaceus*, gecko, *Curcuma zedoaria, Astragalus membranaceus, Atractylodes macrocephala, Poria cocos, Pseudostellaria heterophylla, Panax notoginseng,* and *Hedyotis diffusa*)) in rats with GPL. They found that this decoction could down-regulate ROS, MDA, up-regulate SOD antioxidant activity, and inhibit the invasion and migration of cancer cells, achieve the purpose of repairing damaged gastric mucosal tissues, reversing intestinal metaplasia through regulating the PI3K/AMPK-mTOR-ULK1 pathway.^[128]^ Liu et al confirmed that Shenqi Xiaopi Decoction (Radix Astragali, Radix Codonopsis, Rhizoma Pinelliae, Rhizoma Atractylodis Macrocephalae, Radix Magnoliae Officinalis, Fructus Aurantii Immaturus, Radix Muxiang, Radix Bupleuri, Herba Hedyotis diffusa, cardamom, and Radix Glycyrrhizae) upregulated GSH expression by downregulating MDA, P53 protein, CEA, CA72-4, and IL-8 in the serum of CAG patients.^[129]^ Gu et al found that Ophiopogon japonicus decoction (lentil, raw licorice, *Polygonatum odoratum*, Sha Shen, *Ophiopogon japonicus*, Trichosanthes) can reduce the expression of TNF-α, MDA, and CDX2, increase SOD, epidermal growth factor, MLT, and PGI content, effectively reduce body inflammation, reduce oxidative stress-related factor levels, and achieve good efficacy in clinical treatment of CAG.^[130]^ Some studies have demonstrated that gastritis formula (*Pinellia ternata, Scutellaria baicalensis, Coptis chinensis, Zingiber officinale, Codonopsis pilosula*, and Radix Glycyrrhizae), used to treat CAG patients, resulted in reduced IFN-β, IL-23, and MDA levels, increased SOD antioxidant capacity, decreased inflammatory responses, reduced levels of oxidative stress, and improved pathological signs of the gastric mucosa.^[131]^ In addition, we found that many Chinese herbal prescriptions regulating oxidative stress, such as Shugan Hewei Decoction, Weifuchun, and Xue fu Zhu yu capsule also have demonstrated efficacy in the treatment of CAG.^[132–134]^ These formulations have achieved good therapeutic effects in vivo and in vitro reducing the production of a large number of inflammatory factors as well as oxidative metabolites through the regulation of important signals of oxidative stress and has far-reaching significance in enhancing the repair ability of damaged gastric mucosa and reducing the pathological score of gastric mucosa, laying a certain foundation for the study of the molecular mechanism of such drugs in the treatment of GPL.

#### 2.3.2. Inhibition of inflammatory response, reduction of drug resistance, and improvement of GC prognosis.

An increasing number of studies have confirmed that herbal medicines enhance the efficacy of chemotherapy, radiotherapy, targeted therapy, and immunotherapy for neoplastic diseases. they may also mitigate the adverse effects of these therapies. Banxia Xiexin Decoction (BXD) was first recorded in the book “Miscellaneous Diseases of Typhoid Fever” during the Eastern Han Dynasty. In clinical practice, BXD is used to treat various inflammatory diseases and cancers.^[135]^ After intragastric administration of BXD to GC model rats, MDA, SOD, and GSH contents were regulated, oxidative stress was promoted to induce apoptosis, thereby inhibiting GC cell activity and blocking GC progression. Its mechanism of action is attributed to the inhibition of the Wnt/β-catenin pathway.^[136]^ In addition, Yu et al confirmed that Gancao Ganjiang decoction could inhibit GC progression using network pharmacology and molecular docking technology, and the mechanism may be related to its action on TNF, p53, and other signaling pathways by regulating oxidative stress; however, this needs to be verified by further experiments.^[137]^ You et al reported inhibited NO, IL-6, and C-reaction protein expression in the serum of patients with primary GC after a 2-month treatment with Yiqi Jianpi Jiedu decoction, which indicated that the decoction could obtain a higher short-term efficacy in patients with primary GC, reduce the level of inflammatory factors, and did not increase the incidence of toxicity, rendering it worthy of clinical application.^[138]^ Pan et al reported that Weifuchun capsule extract upregulated Bax and MDA activities, downregulated Bcl-2, SOD, and GSH-Px levels, promoted gastric cancer cell apoptosis, and effectively inhibited gastric cancer progression. In addition, significant changes in these proteins were reported compared to use of 5-FU alone.^[139]^ In addition, She et al confirmed that Pingwei pills were able to upregulate SOD, Nrf2, and HO-1 protein and mRNA expression and enhance cellular antioxidant capacity for effective GC treatment.^[140]^ After SGC-7901 cells were treated with Weiwei’an, the levels of MDA and LDH were inhibited and the activity of SOD was enhanced. It also alleviated oxidative stress and inflammatory factor expression and altered the tumor survival environment. The therapeutic effect of Weiwei’an was achieved by inhibition of the NF-κB pathway.^[141]^ Ye et al found that Tongfu Huoxue decoction effectively promoted the recovery of postoperative gastrointestinal function and improved GC prognosis in patients after radical gastrectomy for gastric cancer to a degree that made it worthy of clinical application. These affects were achieved through downregulating MDA and upregulating SOD activity, thereby protecting intestinal mucosal barrier function and reducing oxidative stress.^[142]^ The above results suggest that these Chinese herbal prescriptions have significant impact in promoting or inhibiting oxidative stress to intervene GC progression and prevent the recurrence and metastasis of tumor cells. In the future, more Chinese herbal prescriptions may become candidates for the treatment of GC (Fig. 2 and Table 2).

**Table 2:** TCM prescriptions in regulating oxidative stress in gastric “inflammation-cancer.”

Name	Models	Biological Effects	Results	Pathway	References
Huatan Xiaoyu Prescription	CAG patients	MLT↑ SOD↑	Improvement of gastrointestinal hormone levels, reduction of body inflammation and inhibition of oxidative stress	----	^[122]^
		NO↓ MDA↓ TNF-α↓			
Shihu Yangwei Decoction	CAG patients	GSH↑ SOD↑	Inhibition of oxidative stress and improvement of clinical symptoms and gastric mucosal pathology	----	^[124]^
		MDA↓ NO↓CRP↓ TNF-α↓ IL-6↓			
Huaganjian	CAG patients	PG I↑ SOD↑	Inhibition of persistent damage to gastric mucosa by oxidative stress and inflammation	----	^[125]^
		PG II↓ IL-1β↓ IL-6↓ IL-17↓ MDA↓			
Weipiling	CAG patients	Nrf2↑ SOD↑ NQO1↑ HO-1↑	Promotion of the activation of Nrf2, improvement of oxidative stress and reverse the gastric “inflammation-cancer” transformation to a certain extent	Nrf2/NQO1/HO-1 pathway	^[127]^
Jianpi Huayu Jiedu Prescription	CAG model rats	SOD↑	Reversion of IM and damage of GECs	PI3K/AMPK-mTOR-ULK1 pathway	^[128]^
		ROS↓ MDA↓			
Shenqi Xiaopi Decoction	CAG patients	GSH-Px↑	Correction of oxidative damage, significantly reduction of tumor markers and p53 protein	----	^[129]^
		MDA↓ P53↓ CEA↓ CA72-4↓ IL-8↓			
Maidong Docoction	CAG patients	SOD↑ EGF↑ MLT↑ PG I↑	Effectively reduce body inflammation and reduce oxidative stress-related factor levels	-----	^[130]^
		TNF-α↓ MDA↓ CDX2↓			
Weiyan Prescription	CAG patients	SOD↑	Inhibit inflammatory reaction, relieve oxidative stress and improve pathological signs of gastric mucosa	----	^[131]^
		IFN-β↓ IL-23↓ MDA↓			
Shugan Hewei Decoction	CAG patients	SOD↑	Inhibition of oxidative stress and inflammatory factor expression, increase of SOD activity, and improvement clinical symptoms	----	^[132]^
		MDA↓ CRP↓ IL-6↓ TNF-α↓ NO↓			
Weifuchun Tablets	CAG patients	SOD↑	Inhibition of oxidative stress, up-regulation of antioxidant enzyme expression, and reduction of gastric mucosa damage	----	^[133]^
		IL-8↓ IL-6↓ TNF-α↓ NO↓			
Xuefu Zhuyu Capsule	CAG patients	SOD↑ CAT↑	Inhibition of oxidative stress and inflammatory damage of gastric mucosa, improvement of pathology	----	^[134]^
		MDA↓ EGF↓			
Banxia xiexin Decoction	GC model rats	MDA↑	promotes oxidative stress to induce apoptosis, thereby inhibiting GC cell activity and blocking GC progression	Wnt/β-catenin pathway	^[136]^
		GSH↓SOD↓			
Gancao ganjiang Decoction	----	VEGFA↓ TNF-α↓	Modulation of oxidative stress to inhibit gastric cancer progression	p53 pathway	^[137]^
Yiqi jianpi jiedu Decoction	GC patients	NO↓ IL-6↓ CRP↓	Reduced adverse effects and increased efficiency	----	^[138]^
Weifuchun Tablet	MGC-803 cells	Bax↑ MDA↑	Promote gastric cancer cell apoptosis and effectively inhibit gastric cancer progression	----	^[139]^
		Bcl-2↓SOD↓GSH-Px↓			
Pingwei Tablet	SGC-7901 cells	SOD↑ Nrf2↑ HO-1↑	Enhancing cellular antioxidant capacity and exerting therapeutic effects on GC	----	^[140]^
Weichang’An	SGC-7901 cells	SOD↑	alleviates oxidative stress and inflammatory expression and alters the tumor survival environment.	NF-κB pathway	^[141]^
		MDA↓ LDH↓			
Tongfu huoxue Decoction	GC patients	SOD↑	protects intestinal mucosal barrier function and reduces oxidative stress, thereby promoting the recovery of postoperative gastrointestinal function and improving GC prognosis	----	^[142]^
		MDA↓			

CAG = chronic atrophic gastritis, CAT = catalase enzyme, CRP = C-Reaction Protein, EGF = epidermal growth factor, GC = gastric cancer, GEC = gastric mucosa epithelial cell, GSH = glutathione, MDA = malondialdehyde, NF-κB = nuclear factor-κB, Nrf2 = nuclear factor erythroid 2-related factor 2, PG = prostaglandin, ROS = reactive oxygen species, SOD = superoxide dismutase, TCM = traditional Chinese medicine.

## 3. Conclusions

CAG is an important stage in the occurrence of GC, although the disease state of CAG has not been clearly defined in TCM. The condition can be best categorized by of “fullness and epigastric pain” according to the clinical manifestations of patients. In TCM theory, the pathogenesis of CAG can be summarized with typical clinical symptoms such as stomachache, bloating, hiccup, and belching and based in the category of “spleen deficiency.” In TCM, the saying is that “vital qi exists in, evil cannot be dry, evil together, its qi will be virtual.” Deficiency of qi in the spleen and stomach of the human body, inability to transport the essence of the water valley, decreased self-regulation ability of Shengqing Jiangzhuo, inability to remove toxic metabolites, and disturbance of the microenvironment in the body lead to the accumulation of damp-heat toxic pathogens in the gastrointestinal tract. Under normal physiological conditions, the human body is in the dynamic balance of yin and yang and life activities proceed in an orderly fashion., If yin or yang cannot be balanced, it will lead to the occurrence of diseases, which is the same as the impact of oxidative stress on the course of CAG and GC elaborated above. Inflammatory factors such as “NF-κB, IL-1, and TNF-α,” like “toxic pathogens” in TCM theory, destroy the normal structure of gastric mucosal tissue and cause persistent damage to the gastric mucosa, and a precancerous microenvironment is formed and accelerate CAG development into GC. Based on this theory, TCM therapy can be used to improve symptoms, regulate the above inflammatory factors and metabolites, improve the antioxidant capacity of the body or inhibit oxidative stress, so that balance (“yin and yang harmony”) is restored, the gastric mucosal pathological score is reduced, the course of gastric “inflammation-cancer,” is inhibited and the incidence of GC and the metastasis of tumor cells is effectively reduced.

A large body of evidence has shown that oxidative stress plays a key role in the course of CAG and GC and can affect patient outcomes and prognosis. Therefore, referring to TCM insight into CAG and GC treatment, there are broad prospects for intervention in the regulation of oxidative stress. In this study, we investigated the mechanism underlying the inhibition of CAG progression and the prevention of GC by regulating key protein targets of oxidative stress. In addition, the active ingredients in TCM used to treat GC can intervene in the progression, drug resistance, and prognosis of GC by bidirectionally regulating oxidative stress. At present, there are some shortcomings in the treatment of CAG and GC using TCM that mediate the oxidative stress pathway. For example, (1) Although relevant controlled studies have shown that TCM can regulate oxidative stress and intervene in CAG and GC progression, there is a lack of multi-sample and multicenter cohort studies. (2) This study mainly focused on a single pathway and target signaling molecules, and the interaction of multiple oxidative stress signaling pathways should be explored in in vivo and in vitro experiments at a later stage to enrich the means of TCM treatment. (3) There have been few studies on the mechanism of action of TCM prescriptions in the treatment of CAG and GC, and such studies should be strengthened in the future to provide more options for TCM in the treatment of these diseases. (4) In the future, more new drugs and more natural products as well as nutraceuticals should be developed using network pharmacology, biological information technology, and metabolomics technology to intervene in the progression of such diseases by intervening in oxidative stress. Taking full advantage of the desirable characteristics of TCM preparations and the experience and information gained from TCM theory will contribute to better health outcomes for patients with CAG and CG.

## Author contributions

**Conceptualization:** Bo Chen, Xinqian Dong, jinlong Zhang, Wei Wang, yujiao Song, Xitong Sun, Kangning Zhao, Zhen Sun.

**Data curation:** Xinqian Dong, jinlong Zhang, Wei Wang, yujiao Song, Xitong Sun, Kangning Zhao, Zhen Sun.

**Formal analysis:** Xinqian Dong, jinlong Zhang, Zhen Sun.

**Visualization:** Bo Chen.

**Writing – original draft:** Bo Chen.

**Writing – review & editing:** Bo Chen.
